# Supplementary material for: Development of Direct Immobilization Technique of Ag Nanoparticles on Resin Substrates Imparting High Antibacterial and Antiviral Activities
Source: Nanomaterials (Basel). 2022 Sep 2;12(17):3046. doi: 10.3390/nano12173046 (PMC9457845; doi:10.3390/nano12173046)
Supplement: Supplementary file 1 [file nanomaterials-12-03046-s001.zip › nanomaterials-1865534-supplementary.pdf]

nanomaterials

Supplementary Materials

Development of Direct Immobilization Technique of Ag Nanoparticles on Resin Substrates Imparting High Antibacterial and Antiviral Activities

Satoshi Seino [1,*], Yuji Ohkubo [1], Tomonari Magara [1], Hiroki Enomoto [1], Eri Nakajima [2], Tomoki Nishida [2], Yasuo Imoto [2], and Takashi Nakagawa [1]

[1] Graduate School of Engineering, Osaka University, 2-1 Yamadaoka, Suita, Osaka 565-0871, Japan
[2] Japan Textile Products Quality and Technology Center, 5-7-3, Shimoyamate-dori, Chuo-ku, Kobe-city 650-0011, Hyogo, Japan
* Correspondence: seino@mit.eng.osaka-u.ac.jp; Tel.: +81-6-6879-7887

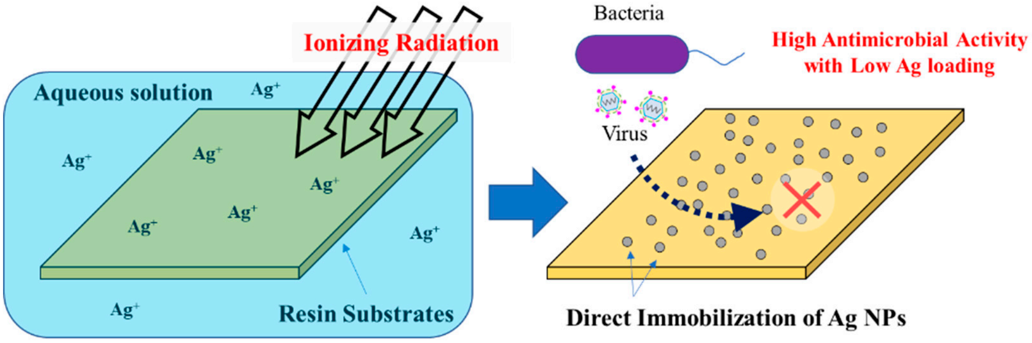

Figure S1. Schematic illustration of this work. Ag nanoparticles with high antimicrobial activities were directly immobilized on resin substates by using chemical reactions induced by ionizing radiation.

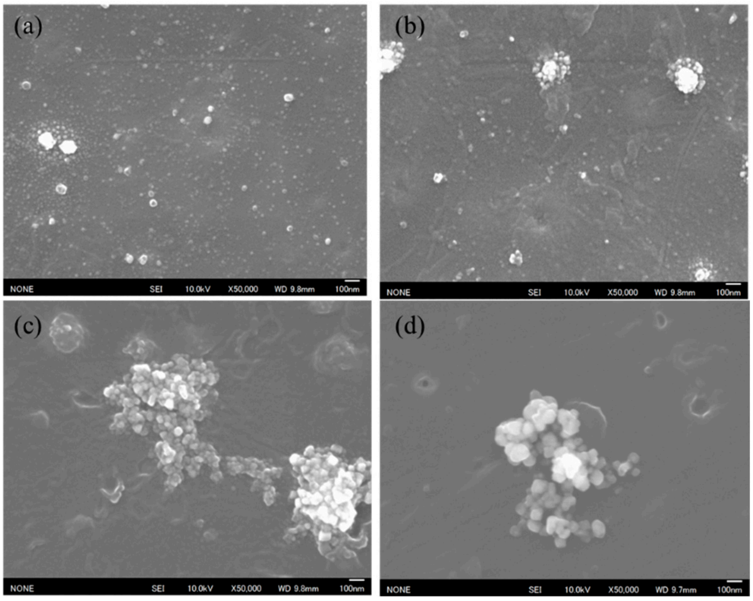

Figure S2. SEM images of Ag/ABS samples synthesized by using radiation observed at high magnification (×50,000).
(a) Gamma-ray, w/o 2-prop., (b) Gamma-ray, w/ 2-prop. (10 vol.%),
(c) Electron Beam, w/o 2-prop., (d) Electron Beam w/ 2-prop. (10 vol.%).

Table S1. Antibacterial activity of Ag/ABS samples against *E.coli*.

**Samples**		**Viable cell count**[*1]
PE Film (Control)	Immediately after inoculation	4.05
		4.06
		4.06
	After 24 h	6.07
		6.06
		6.10
ABS (Control)	After 24 h	5.67
		5.67
		5.71
Ag/ABS (Gamma-ray, w/o 2prop.)	After 24 h	< -0.20
		< -0.20
		< -0.20
Ag/ABS (Electron Beam, w/o 2prop.)	After 24 h	< -0.20
		< -0.20
		< -0.20

[*1]The common logarithm for the number of bacterial colonies.

Table S2 Antibacterial activity of Ag/resin-plate samples synthesized by gamma-ray against *E.coli*.

**Samples**		**Viable cell count**[*1]
PE Film (Control)	Immediately after inoculation	4.07
		4.04
		4.05
	After 24 h	5.84
		5.94
		5.75
PE (Control)	After 24 h	3.71
		5.90
		4.80
Ag/PE (w/o 2-prop.)	After 24 h	< -0.20
		< -0.20
		< -0.20
Ag/PE (w/ 2prop. 10 vol.%)	After 24 h	< -0.20
		< -0.20
		< -0.20
PP (Control)	After 24 h	5.76
		0.45
		3.23
Ag/PP (w/o 2-prop.)	After 24 h	< -0.20
		< -0.20
		< -0.20
Ag/PP (w/ 2prop. 10 vol.%))	After 24 h	< -0.20
		< -0.20
		< -0.20
PVC (Control)	After 24 h	5.46
		5.65
		5.55
Ag/PVC (w/o 2-prop.)	After 24 h	< -0.20
		< -0.20
		< -0.20
Ag/PVC (w/ 2prop. 10 vol.%))	After 24 h	< -0.20
		< -0.20
		< -0.20
PC (Control)	After 24 h	5.61
		5.67
		5.65
Ag/PC (w/o 2-prop.)	After 24 h	< -0.20
		< -0.20
		< -0.20
Ag/PC (w/ 2prop. 10 vol.%))	After 24 h	< -0.20
		< -0.20
		< -0.20

[*1]The common logarithm for the number of bacterial colonies.

Table S3. Antiviral activity of Ag/ABS samples synthesized by electron beam against Influenza-A virus.

Samples		Common logarithm of infectivity titre value (PFU/cm^2)
ABS (Control)	Immediately after inoculation	5.46
		5.43
		5.47
	After 24 h	4.57
		4.64
		4.67
Ag/ABS (EB, w/o 2prop.)	After 24 h	4.09
		1.54
		3.01
Ag/ABS (EB, w/ 2prop. 1vol.%)	After 24 h	4.43
		0.97
		2.574
Ag/ABS (EB, w/ 2prop. 10 vol.%)	After 24 h	< 0.80
		2.61
		1.19

Test Virus; Influenza A virus (H3N2) (A/Hong Kong/8/68;TC adapted ATCC 1679)

Table S4. Antiviral activity of Ag/ABS samples synthesized by gamma-ray against SARS-CoV-2.

Samples		Common logarithm of infectivity titre value (PFU/cm^2)
ABS (Control)	Immediately after inoculation	5.67
		5.61
		5.67
	After 24 h	4.70
		5.08
		4.67
Ag/ABS (Gamma-ray, w/o 2prop.)	After 24 h	0.97
		1.10
		1.10

Test Virus; SARS-CoV-2, (NIID isolate : JPN/TY/WK-521)
